# Dynamic changes in liver function parameters in patients with coronavirus disease 2019: a multicentre, retrospective study

**DOI:** 10.1186/s12879-021-06572-z

**Published:** 2021-08-16

**Authors:** Qing-Lei Zeng, Zu-Jiang Yu, Fanpu Ji, Guang-Ming Li, Guo-Fan Zhang, Jiang-Hai Xu, Wan-Bao Lin, Guo-Qiang Zhang, Guo-Tao Li, Guang-Lin Cui, Fu-Sheng Wang

**Affiliations:** 1 Department of Infectious Diseases and Hepatology, The First Affiliated Hospital of Zhengzhou University, No. 1 Eastern Jianshe Road, Zhengzhou, 450052 Henan China; 2 Department of Infectious Diseases, The Second Affiliated Hospital of Xi’an Jiaotong University, Xi’an, Shaanxi China; 3 Key Laboratory of Environment and Genes Related To Diseases, Ministry of Education of China, Xi’an Jiaotong University, Xi’an, Shaanxi China; 4 Department of Infectious Diseases, The Sixth People’s Hospital of Zhengzhou City, Zhengzhou, Henan China; 5 The Department of Infectious Diseases, The First Affiliated Hospital of Nanyang Medical College, Nanyang, Henan China; 6 The Department of Infectious Diseases, The Fifth People’s Hospital of Anyang City, Anyang, Henan China; 7 The Department of Infectious Diseases, Xinyang Central Hospital, Xinyang, Henan China; 8 The Department of Infectious Diseases, Luoyang Central Hospital, Luoyang, Henan China; 9 Department of Clinical Laboratory, The First Affiliated Hospital of Zhengzhou University, Zhengzhou, Henan China; 10 Treatment and Research Center for Infectious Diseases, The Fifth Medical Center of Chinese PLA General Hospital, National Clinical Research Centre for Infectious Diseases, No. 100 Western 4th Middle Ring Road, Fengtai District, Beijing, China

**Keywords:** Aalanine aminotransferase, Aspartate aminotransferase, Coronavirus disease 2019, Dynamic changes, Liver function, Liver injury, Severe acute respiratory syndrome coronavirus 2

## Abstract

**Background:**

Liver injuries have been reported in patients with coronavirus disease 2019 (COVID-19). This study aimed to investigate the clinical role played by severe acute respiratory syndrome coronavirus 2 (SARS-CoV-2).

**Methods:**

In this multicentre, retrospective study, the parameters of liver function tests in COVID-19 inpatients were compared between various time-points in reference to SARS-CoV-2 shedding, and 3 to 7 days before the first detection of viral shedding was regarded as the reference baseline.

**Results:**

In total, 70 COVID-19 inpatients were enrolled. Twenty-two (31.4%) patients had a self-medication history after illness. At baseline, 10 (14.3%), 7 (10%), 9 (12.9%), 2 (2.9%), 15 (21.4%), and 4 (5.7%) patients already had abnormal alanine aminotransferase (ALT), aspartate aminotransferase (AST), gamma-glutamyl transferase (GGT), alkaline phosphatase (ALP), albumin, and total bilirubin (TBIL) values, respectively. ALT and AST abnormal rates and levels did not show any significant dynamic changes during the full period of viral shedding (all *p* > 0.05). The GGT abnormal rate (*p* = 0.008) and level (*p* = 0.033) significantly increased on day 10 of viral shedding. Meanwhile, no simultaneous significant increases in abnormal ALP rates and levels were observed. TBIL abnormal rates and levels significantly increased on days 1 and 5 of viral shedding (all *p* < 0.05). Albumin abnormal decrease rates increased, and levels decreased consistently from baseline to SARS-CoV-2 clearance day (all *p* < 0.05). Thirteen (18.6%) patients had chronic liver disease, two of whom died. The ALT and AST abnormal rates and levels did not increase in patients with chronic liver disease during SARS-CoV-2 shedding.

**Conclusions:**

SARS-CoV-2 does not directly lead to elevations in ALT and AST but may result in elevations in GGT and TBIL; albumin decreased extraordinarily even when SARS-CoV-2 shedding ended.

## Background

Since December 2019, coronavirus disease 2019 (COVID-19) caused by severe acute respiratory syndrome coronavirus 2 (SARS-CoV-2) has affected nearly all countries throughout the world in the past nine months [1]. On March 11, 2020, COVID-19 was characterized as a pandemic by the World Health Organization (WHO). As of September 25, 2020, a total of 32,110,656 people have been infected with SARS-CoV-2 worldwide, with a mortality rate of approximately 3.1% (980,031 deaths) [1]. However, no effective drugs are clinically approved because of the absence of evidence [2], despite we preliminarily found that convalescent plasma treatment can halt SARS-CoV-2 shedding [3].

Recently, several studies have described the epidemiological and clinical characteristics of patients with COVID-19 in China [4, 5]. Of these studies, liver injury has been reported in 16.1% to 53.1% of patients, which raised concern about the relationship between SARS-CoV-2 and liver impairment [6]. It is likely that the liver dysfunction seen in COVID-19 is multifactorial, such as direct viral damage, inflammatory response, hypoxia, microthrombotic events, and drug-induced liver injury (DILI) [7, 8]. SARS-CoV-2 enters the host via the angiotensin-converting enzyme 2 (ACE2) receptor, Chai et al. found cholangiocytes highly express ACE2, indicating that the liver could be a potential target for SARS-CoV-2 [9]. Notably, the details of liver injury occurrence were confusing in these studies. First, liver injury can occur before the illness and viral shedding, at various time-points during the illness and viral shedding, and on or after the day of viral clearance. At different time-points, abnormal rates and levels of liver injury may vary. Additionally, many parameters have been employed to reflect liver injury, and most of these studies only report one or two parameters, such as alanine aminotransferase (ALT) or aspartate aminotransferase (AST), which hinders observation of the panorama of liver injury. Furthermore, no dynamic changes in liver function test parameters to reflect liver injury have been reported. These limitations prevent us from exploring the actual relationship between SARS-CoV-2 and liver injury. In the current study, we aimed to investigate the role of SARS-CoV-2 in liver injury through a multicentre, retrospective cohort with full continuous data on liver function test parameters and clinical course, especially full duration of viral shedding.

## Methods

### Data sources

According to arrangements by the Chinese government, all COVID-19 patients were admitted centrally to designated local hospitals. This retrospective, multicentre, observational study included 6 designated referral hospitals in Henan Province, which is near Hubei Province and had the third largest number of COVID-19 patients in China [10]. We retrospectively collected and analysed the epidemiological, clinical, laboratory, virologic, management, and outcome data on patients with laboratory-confirmed SARS-CoV-2 infection from electronic medical records by using a predesigned case report form. The COVID-19 patients were enrolled during the main epidemic period from January 20 to February 29, 2020 (before January 19 and after March 1, nearly no and few patients were identified in Henan Province, respectively), and clinical outcomes were followed up until March 26, 2020.

### Definition of laboratory-confirmed COVID-19

Laboratory-confirmed COVID-19 was diagnosed according to WHO interim guidance [11]. Laboratory confirmation of SARS-CoV-2 infection was performed in the Centers for Disease Control and Prevention (CDCs) of the government, i.e., the Henan Provincial CDC; the CDCs of Xinyang, Nanyang, Anyang, and Luoyang cities of Henan province; and Shaanxi Provincial CDC. Real-time reverse transcriptase polymerase chain reaction (RT-PCR) tests for SARS-CoV-2 RNA were performed using nasopharyngeal swabs (Novel Coronavirus PCR Fluorescence Diagnostic Kit, BioGerm Medical Biotechnology) [3]. The specimen pretreatment, and RNA extraction procedures; RT-PCR conditions; and results interpretation strictly followed the manufacturers’ instructions.

### Main criterion of hospital discharge or recovery of COVID-19 patients

The criteria for hospital discharge (recovery) were the same as those described by Lan et al. [12]. Briefly, the criteria for hospital discharge were mainly based on recovery of the symptoms and signs, undetectable SARS-CoV-2 RNA, and resolution of lung inflammation (such as ground-glass opacities and/or consolidations). The SARS-CoV-2 RNA negativity was confirmed at least two times, with an interval of more than 48 h.

### Inclusion, exclusion, and grouping criteria for the current study

**Fig. 1:**
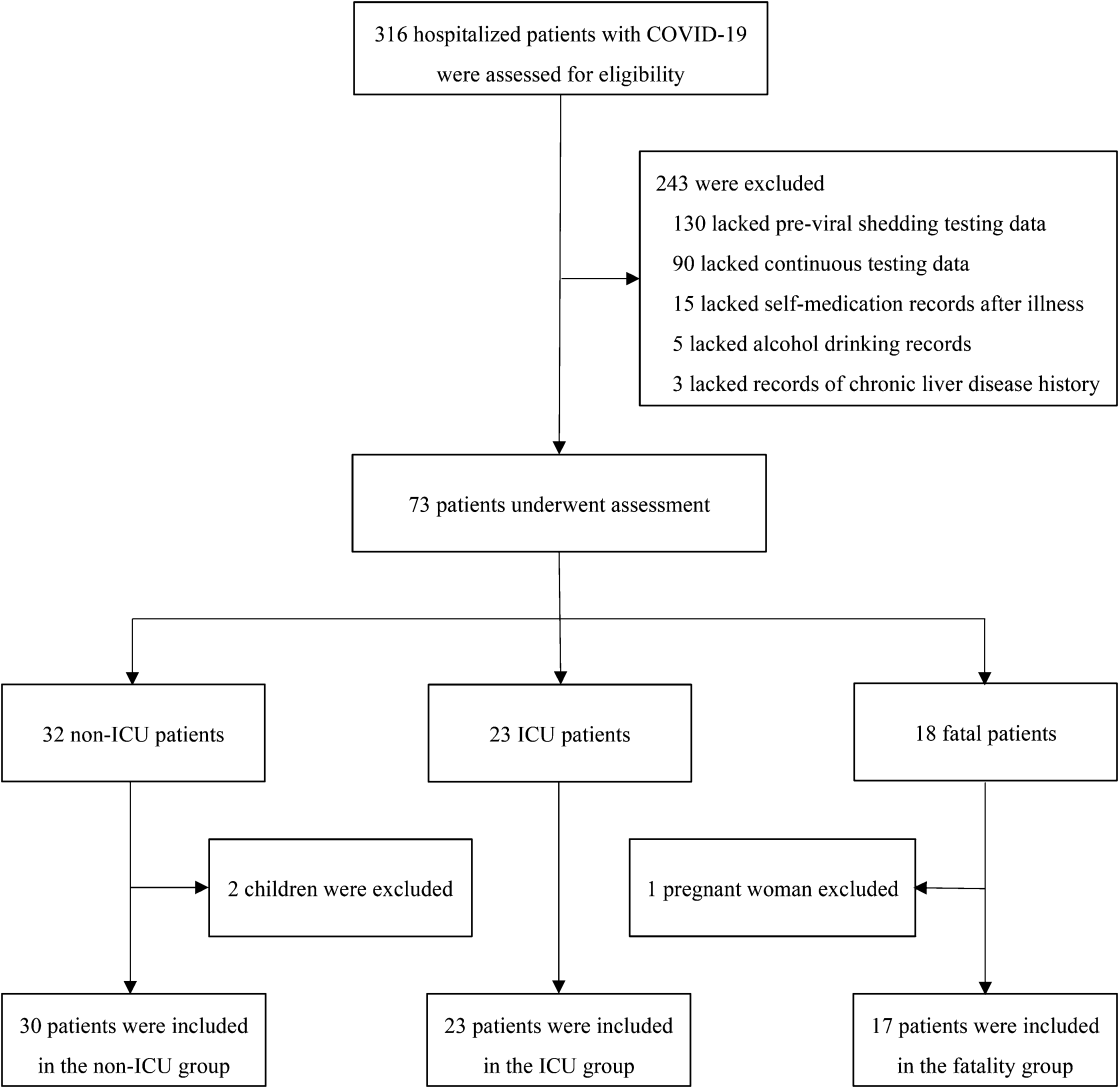
Flow diagram of the study enrolment. COVID-19, coronavirus diseases 2019; ICU, intensive care unit

All male and nonpregnant female patients 18 years of age or older were eligible for inclusion if they had full liver function test parameter records and complete clinical course data during the abovementioned study duration (Fig. 1), which indicated that the patients had detailed data from illness to discharge (recovery) or death. Exclusion criteria included patients who did not meet the abovementioned inclusion criteria, lacked medical records of relevant self-medication information, alcohol drinking condition, or previous diagnosis of chronic liver disease. The COVID-19 illness severity was defined according to the Chinese management guidelines for COVID-19 (version 6.0) [13]. Based on the severity and clinical outcomes, the patients in the current study were divided into 3 groups, i.e., non-intensive care unit (ICU), ICU, and fatality groups.

### Liver function test parameters and abnormal rates

The liver function test parameters in the current study included serum ALT, AST, gamma-glutamyl transferase (GGT), alkaline phosphatase (ALP), ALB, and total bilirubin (TBIL). An abnormal rate was defined as the proportion of a liver function test parameter more than the upper limit of the normal range (applied to ALT, AST, GGT, ALT, and TBIL) or less than the lower limit of the normal range (applied to ALB only) in a group of patients. The upper limit of the normal range for the most important parameter to reflect liver injury, i.e., ALT, was defined as 40 U/L according to the Asian Pacific Association for the Study of the Liver guidelines committee [14].

### Study timepoints

Using SARS-CoV-2 shedding as the reference, 6 time-points were investigated in the current study, i.e., 3 to 7 days before the first detection of viral shedding (day-[3–7]), the first day of viral shedding (day 1), the fifth day of viral shedding (day 5), the tenth day of viral shedding (day 10), the fifteenth day of viral shedding (day 15), and the day of SARS-CoV-2 clearance (day clearance). Day-[3–7] was defined as the reference baseline.

### Study outcomes

The study outcomes were abnormal rates and detailed levels of liver function test parameters at various time-points in reference to viral shedding in different groups of COVID-19 patients, including a separate subgroup of patients with chronic liver disease. The dynamic abnormal rates and levels of liver function test parameters were investigated to analyse the potential relationship between SARS-CoV-2 and liver injury as well as the potential role of SARS-CoV-2 in patients with chronic liver disease.

### Statistical analysis

Continuous and categorical variables are presented as the median (interquartile range), mean ± standard deviation, and n (%) where appropriate. A Mann–Whitney U test was used to compare differences in measurement data between two independent groups, and a Wilcoxon signed rank test was used to compare differences in measurement data between various timepoints for the same group. Chi-squared test or Fisher’s exact test were used to compare rate differences between various subgroups. Analyses were carried out using SPSS statistical software, version 25.0 (IBM, Chicago, IL, USA). A *p* value of < 0.05 was set as the threshold for statistical significance.

## Results

### Demographic and clinical characteristics

A total of 316 hospitalized, laboratory-confirmed COVID-19 patients were assessed for eligibility (Fig. 1), and 70 patients were eventually enrolled according to the inclusion and exclusion criteria. Among them, 30 asymptomatic, mild, and common patients were placed in the non-ICU group; 23 severe and critical surviving patients were placed in the ICU group; and 17 critical, non-surviving patients were placed in the fatality group. The demographic and clinical characteristics of these patients are presented in Table 1.

**Table 1:** Demographic and clinical characteristics of patients with COVID-19

	Total (n = 70)	Non-ICU (n = 30)	ICU (n = 23)	Fatality (n = 17)
Age, years	56.5 (41–73)	46 (31–65.5)	57 (44–66)	73 (63–79)
Sex, Male	46 (65.7)	20 (66.7)	13 (56.5)	13 (76.5)
Viral shedding period, days	12 (9–16.3)	9 (6–11.3)	13 (11–16)	19 (12.5–21)^a^
Chronic comorbidities				
Hypertension	18 (25.7)	8 (26.7)	7 (30.4)	3 (17.6)
Diabetes	13 (18.6)	3 (10)	5 (16.7)	5 (29.4)
Cardiovascular diseases	7 (10)	2 (6.7)	3 (13)	2 (11.8)
Chronic kidney disease	4 (5.7)	20	3 (13)	1 (5.9)
Respiratory system diseases	1 (1.4)	0 (0)	0 (0)	1 (5.9)
Chronic liver diseases	13 (18.6)	6 (20)	5 (16.7)	2 (11.8)
Chronic hepatitis B	5 (7.1)	1 (3.3)	3 (13)	1 (5.9)
Alcoholic liver disease	5 (7.1)	5 (16.7)	0 (0)	0 (0)
Fatty liver disease	2 (2.9)	0 (0)	1 (4.3)	1 (5.9)
Chronic hepatitis C	1 (1.4)	0 (0)	1 (4.3)	0 (0)
HBV markers				
HBsAg/HBeAb/HBcAb (+)	5 (7.1)	1 (3.3)	3 (13)	1 (5.9)
HBsAb/HBeAb/HBcAb (+)	38 (54.3)	10 (33.3)	15 (65.2)	13 (76.5)
All HBV markers (−)	11 (15.7)	6 (20)	3 (13)	2 (11.8)
Sole HBsAb (+)	7 (10)	6 (20)	0 (0)	1 (5.9)
Sole HBcAb (+)	4 (5.7)	3 (10)	1 (4.3)	0 (0)
HBeAb/HBcAb (+)	3 (4.3)	2 (6.7)	1 (4.3)	0 (0)
HBsAb/HBcAb (+)	2 (2.9)	2 (6.7)	0 (0)	0 (0)
Self-medication after illness	22 (31.4)	8 (26.7)	6 (26.1)	8 (47.1)
Traditional Chinese medicine	14 (20)	6 (6.7)	4 (17.4)	4 (23.5)
Acetaminophen	4 (5.7)	2 (6.7)	1 (4.3)	1 (5.9)
Levofloxacin/Moxifloxacin	4 (5.7)	0 (0)	1 (4.3)	3 (17.6)
Treatment during hospitalization				
Symptomatic treatment	62 (88.6)	22 (73.3)	23 (100)	17 (100)
Antivirals^b^	56 (80)	20 (66.7)	19 (82.6)	17 (100)
Antibiotics^c^	36 (51.4)	8 (26.7)	11 (47.8)	17 (100)
Traditional Chinese medicine	12 (17.1)	3 (10)	5 (21.7)	4 (23.5)
Immunoglobulin	20 (28.6)	0 (0)	7 (30.4)	13 (76.5)
Glucocorticoid	17 (24.3)	0 (0)	5 (21.7)	12 (70.6)
High-flow oxygen	35 (50)	0 (0)	18 (78.3)	17 (100)
Mechanical ventilation	17 (24.3)	0 (0)	3 (13)	14 (82.4)
CRRT	11 (15.7)	0 (0)	0 (0)	11 (64.7)

Data are presented as median (interquartile range) or n (%)

^a^The viral shedding period in the fatality group was calculated from the beginning of detectable SARS-CoV-2 to the discontinuation of viral shedding or to the death date, even though viral shedding continued at the time of fatality

^b^Antiviral agents mainly included oseltamivir, interferon α (aerosol inhalation), and lopinavir/ritonavir

^c^Antibiotics mainly included levofloxacin and moxifloxacin for all groups and meropenem, biapenem, vancomycin, and tigecycline for the fatality group

In total, the median age was 56.5 (41–73) years, and age gradually increased from the non-ICU to the ICU and fatality groups. A total of 65.7% (46) of patients were male. The median viral shedding durations were 9 (6–11.3), 13 (11–16), and 19 (12.5–21) days in the non-ICU, ICU, and fatality groups, respectively. The most common comorbidities were hypertension (18, 25.7%), diabetes (13, 18.6%), and cardiovascular disease (7, 10%). Thirteen (18.6%) patients were previously diagnosed with chronic liver disease; 5 patients had hepatitis B e antigen (HBeAg)-negative chronic hepatitis B, and 2 of them received entecavir treatment; 5 patients had alcoholic liver disease; 2 had fatty liver disease; and 1 had chronic hepatitis C. Notably, 1 patient with chronic hepatitis B and 1 patient with fatty liver disease eventually died. It is important to note that 38 (54.3%) patients had a combination of three HBV antibodies, i.e., antibodies to surface antigen (HBsAb), e antigen (HBeAb), and core antigen (HBcAb); more importantly, the percentages of this combination were gradually increased in the three groups (non-ICU [33.3%] vs ICU [65.2%], *p* = 0.001; non-ICU [33.3%] vs fatality [76.5%], *p* = 0.004). Before visit or admission to the hospital, 22 (31.4%) patients had a history of self-medication after illness onset. During hospitalization, symptomatic treatment (62, 88.6%), antivirals (56, 80%), and antibiotics (36, 51.4%) were the most common treatment strategies.

### Dynamic abnormal rates of liver function test parameters between the three groups

The dynamic abnormal rates of liver function test parameters between the three groups are presented in Table 2. Notably, 10 (14.3%) and 7 (10%) patients had ALT and AST values, respectively, more than the upper limit of normal ranges on day −(3–7), and these abnormal rates did not increase on day 1. Additionally, no significant differences in ALT and AST abnormal rates were observed between the three groups on day −(3–7) and day 1. On day 5, the AST abnormal rate increased in the fatality group (non-ICU [3.8%] vs fatality [37.5%], *p* = 0.008; ICU [8.7%] vs fatality [37.5%], *p* = 0.045), and the same phenomenon was observed on day clearance (non-ICU [0] vs fatality [44.4%], *p* = 0.002; ICU [8.7%] vs fatality [44.4%], *p* = 0.038). Meanwhile, on day 5, the TBIL abnormal rate increased in the fatality group (ICU [8.7%] vs fatality [43.8%], *p* = 0.019), and the same phenomenon also observed on day 10 (non-ICU [0] vs fatality [69.2%], *p* = 0.005; ICU [0] vs fatality [69.2%], *p* < 0.001) and on day clearance (non-ICU [10%] vs fatality [55.6%], *p* = 0.009; ICU [8.7%] vs fatality [55.6%], *p* = 0.010). Although the GGT and ALP abnormal rates accounted for 12.9%-34.1% and 1.4%-10.8%, respectively, at various time-points in all patients, no significant differences were found between the three groups, with the exception of the ALP abnormal rate, which significantly increased on day 10 in the fatality group (ICU [0] vs fatality [30.8%], *p* = 0.017). Additionally, no significant differences in abnormal rates for ALB were observed between the three groups, with the exception of abnormally decreased rates that increased on day 5 in the ICU group (non-ICU [26.9%] vs ICU [56.5%], *p* = 0.035).

**Table 2:** Dynamic abnormal rates of liver function test parameters in different groups of patients with COVID-19

	Total (n = 70)	Non-ICU (n = 30)	ICU (n = 23)	Fatality (n = 17)	*p*1	*p*2	*p*3
Day 3–7 before viral shedding							
ALT increased (0–40 U/L)	10 (14.3)	5 (16.7)	3 (13)	2 (11.8)	1.000	1.000	1.000
AST increased (0–40 U/L)	7 (10)	2 (6.7)	3 (13)	2 (11.8)	0.642	0.613	1.000
GGT increased (0–58 U/L)	9 (12.9)	5 (16.7)	2 (8.7)	2 (11.8)	0.685	1.000	1.000
ALP increased (40–130 U/L)	2 (2.9)	1 (3.3)	0 (0)	1 (5.9)	1.000	1.000	0.425
ALB decreased (35–55 g/L)	15 (21.4)	6 (20)	4 (17.4)	5 (29.4)	1.000	0.709	0.605
TBIL increased (0–25 μmol/L)	4 (5.7)	0 (0)	2 (8.7)	2 (11.8)	0.184	0.126	1.000
During viral shedding, on day 1							
ALT increased (0–40 U/L)	8 (11.4)	4 (13.3)	1 (4.3)	3 (17.6)	0.374	0.692	0.294
AST increased (0–40 U/L)	7 (10)	3 (10)	1 (4.3)	3 (17.6)	0.624	0.653	0.294
GGT increased (0–58 U/L)	15 (21.4)	8 (26.7)	3 (13)	4 (23.5)	0.384	1.000	0.432
ALP increased (40–130 U/L)	1 (1.4)	1 (3.3)	0 (0)	0 (0)	1.000	1.000	-
ALB decreased (35–55 g/L)	30 (42.9)	10 (33.3)	10 (43.5)	10 (58.8)	0.450	0.089	0.337
TBIL increased (0–25 μmol/L)	12 (17.1)	5 (16.7)	3 (13)	4 (23.5)	1.000	0.850	0.432
During viral shedding, on day 5							
ALT increased (0–40 U/L)	11/65 (16.9)	5/26 (19.2)	2 (8.7)	4/16 (25)	0.424	0.956	0.205
AST increased (0–40 U/L)	9/65 (13.8)	1/26 (3.8)	2 (8.7)	6/16 (37.5)	0.594	0.008	0.045
GGT increased (0–58 U/L)	14/65 (21.5)	7/26 (26.9)	5 (21.7)	2/16 (12.5)	0.674	0.472	0.678
ALP increased (40–130 U/L)	7/65 (10.8)	2/26 (7.7)	1 (4.3)	4/16 (25)	1.000	0.180	0.139
ALB decreased (35–55 g/L)	29/65 (44.6)	7/26 (26.9)	13 (56.5)	9/16 (37.5)	0.035	0.057	1.000
TBIL increased (0–25 μmol/L)	14/65 (21.5)	5/26 (19.2)	2 (8.7)	7/16 (43.8)	0.424	0.175	0.019
During viral shedding, on day 10							
ALT increased (0–40 U/L)	8/41 (19.5)	0/8 (0)	4/20 (20)	4/13 (30.8)	0.295	0.131	0.681
AST increased (0–40 U/L)	6/41 (14.6)	0/8 (0)	2/20 (10)	4/13 (30.8)	1.000	0.131	0.182
GGT increased (0–58 U/L)	14/41 (34.1)	3/8 (37.5)	7/20 (35)	4/13 (30.8)	1.000	1.000	1.000
ALP increased (40–130 U/L)	4/41 (9.8)	0/8 (0)	0/20 (0)	4/13 (30.8)	-	0.131	0.017
ALB decreased (35–55 g/L)	23/41 (56.1)	5/8 (62.5)	10/20 (50)	8/13 (61.5)	0.686	1.000	0.722
TBIL increased (0–25 μmol/L)	9/41 (22)	0/8 (0)	0/20 (0)	9/13 (69.2)	-	0.005	< 0.001
On the day of viral clearance							
ALT increased (0–40 U/L)	7/62 (11.3)	3 (10)	2 (8.7)	2/9 (22.2)	1.000	0.572	0.557
AST increased (0–40 U/L)	6/62 (9.7)	0 (0)	2 (8.7)	4/9 (44.4)	0.184	0.002	0.038
GGT increased (0–58 U/L)	19/62 (30.6)	9 (30)	8 (34.8)	2/9 (22.2)	0.712	0.974	0.681
ALP increased (40–130 U/L)	1/62 (1.6)	0 (0)	1 (4.3)	0/9 (0)	0.434	-	1.000
ALB decreased (35–55 g/L)	32/62 (51.6)	13 (43.3)	14 (60.9)	5/9 (55.6)	0.206	0.706	1.000
TBIL increased (0–25 μmol/L)	10/62 (16.1)	3 (10)	2 (8.7)	5/9 (55.6)	1.000	0.009	0.010

Data are presented as n (%) or n/N (%), where N is the total number of cases with available data. Data were unavailable in some patients because viral shedding discontinuation occurred before this timepoint or viruses were not cleared until death (fatality group). The corresponding normal ranges and units of the liver function test parameters are presented in parentheses; increased indicates over the upper limit of the normal range, and decreased indicates below the lower limit of the normal range. *p*1: non-ICU vs ICU groups, *p*2: non-ICU vs fatality, *p*3: ICU vs fatality

### Dynamic abnormal rates and levels of liver function test parameters in all patients

**Fig. 2:**
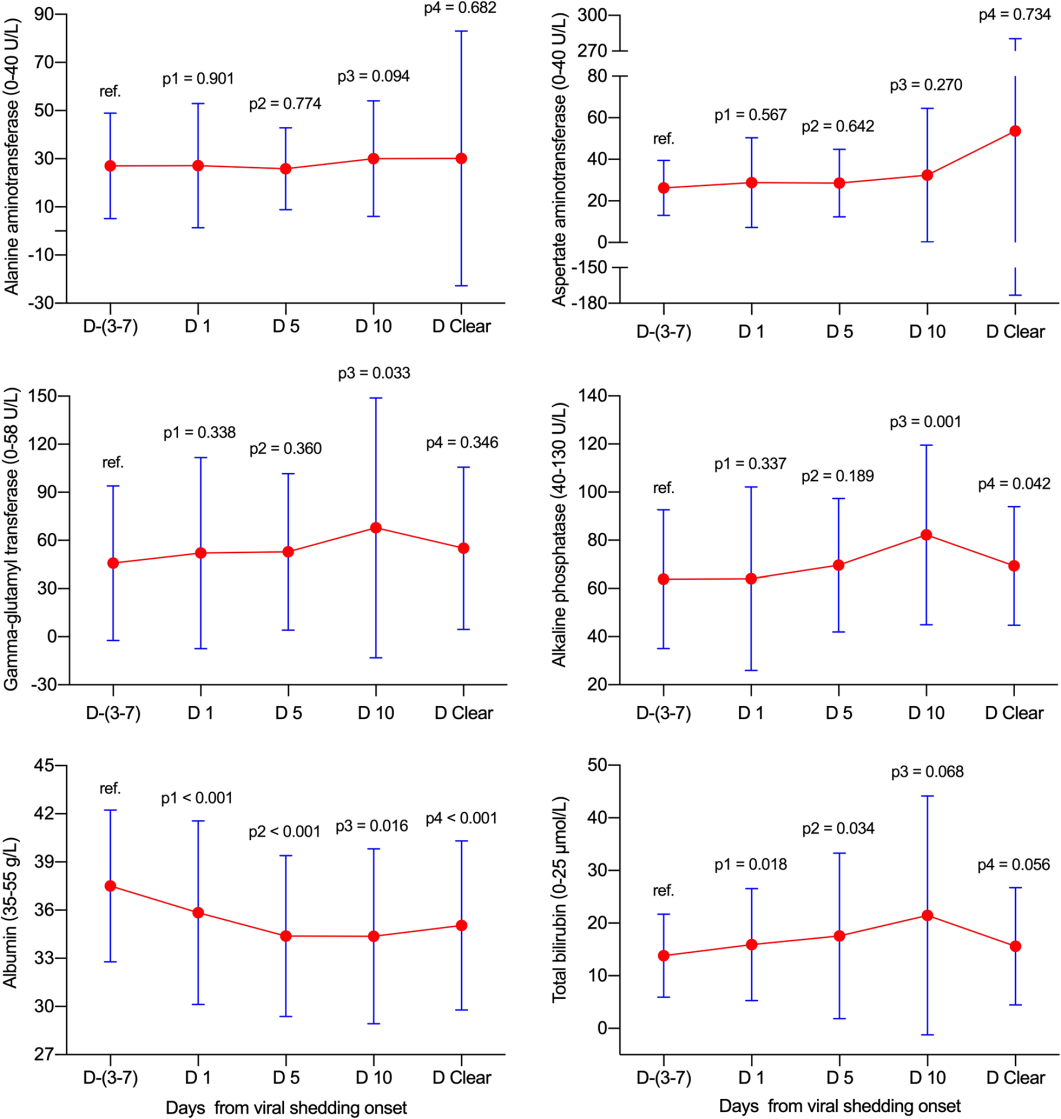
Dynamic levels of liver function test parameters in all patients. Data were presented as mean ± standard deviation. D −(3–7): n = 70; D 1: n = 70; D 5: n = 65; D 10: n = 41; D Clear: n = 62. D, day; ref., reference

In total, no significant elevations in abnormal rates and levels for ALT and AST were found at various time-points in reference to viral shedding (all *p* > 0.05, Table 3 and Fig. 2). Meanwhile, the GGT abnormal rate (*p* = 0.008) and level (*p* = 0.033) increased on day 10, and ALP levels increased on day 10 (*p* = 0.001) and on day clearance (*p* = 0.042) without matching with abnormal rates (both *p* > 0.05). Notably, the ALB abnormal rates increased, and levels decreased gradually from day −(3–7) to day clearance (all *p* < 0.05). Additionally, the TBIL abnormal rates and levels significantly increased on days 1 and 5 (all *p* < 0.05), and no matched significant differences were observed on day 10 and day clearance.

**Table 3:** Dynamic abnormal rates and levels of liver function test parameters between various viral shedding timepoints in patients with COVID-19

	Day −(3–7) (n = 70)	Day 1 (n = 70)	Day 5 (n = 65)	Day 10 (n = 41)	Day-clearance (n = 62)	*p*1	*p*2	*p*3	*p*4
ALT (0–40 U/L)	21 (13–27.3)	21 (14–28)	21 (15–31)	22 (17–34.5)	20 (13.8–29)	0.901	0.774	0.094	0.682
> 40 U/L	10 (14.3)	8 (11.4)	11 (16.9)	8 (19.5)	7 (11.3)	0.614	0.673	0.471	0.608
AST (0–40 U/L)	23 (19–32)	24 (19–32)	24 (19–33)	25 (17–33.5)	22.4 (16–32)	0.567	0.642	0.270	0.734
> 40 U/L	7 (10)	7 (10)	9 (13.8)	6 (14.6)	6 (9.7)	-	0.490	0.669	0.951
GGT (0–58 U/L)	33.5 (22–48)	33 (23.8–51)	33.5 (23–58)	43 (26–67)	33 (24–66.8)	0.338	0.360	0.033	0.346
> 58 U/L	9 (12.9)	15 (21.4)	14 (21.5)	14 (34.1)	19 (30.6)	0.178	0.180	0.008	0.013
ALP (40–130 U/L)	59.5 (53.8–67)	57.5 (47–73.3)	64 (50.3–80)	73 (57–93)	67 (51–78)	0.337	0.189	0.001	0.042
> 130 U/L	2 (2.9)	1 (1.4)	7 (10.8)	4 (9.8)	1 (1.6)	1.000	0.088	0.191	1.000
ALB (35–55 g/L)	37 (34–40.1)	36 (31.3–40)	35 (31.3–38)	34.2 (31.4–38)	34.5 (31.7–37.2)	< 0.001	< 0.001	0.016	< 0.001
< 35 g/L	15 (21.4)	30 (42.9)	29 (44.6)	23 (56.1)	32 (51.6)	0.007	0.004	< 0.001	< 0.001
TBIL (0–25 μmol/L)	11.5 (8–18.6)	12.2 (9.3–23.5)	14 (9–21.5)	14.8 (8.5–27.7)	11.8 (8.2–21.1)	0.018	0.034	0.068	0.056
> 25 μmol/L	4 (5.7)	12 (17.1)	14 (21.5)	9 (22)	10 (16.1)	0.034	0.007	0.024	0.052

Data are presented as median (interquartile range) and n (%). The corresponding normal ranges and units of the liver function test parameters are presented in parentheses. Data were unavailable for patients on day 5 (n = 5), 10 (n = 29), and at viral clearance (n = 8) because viral shedding discontinuation occurred before this timepoint or because the virus was not cleared until death (day-clearance timepoint). *p*1: day −(3–7) vs day 1, *p*2: day −(3–7) vs day 5, *p*3: day −(3–7) vs day 10, *p*4: day −(3–7) vs day clearance

### Dynamic levels of liver function test parameters in non-ICU and ICU groups

**Fig. 3:**
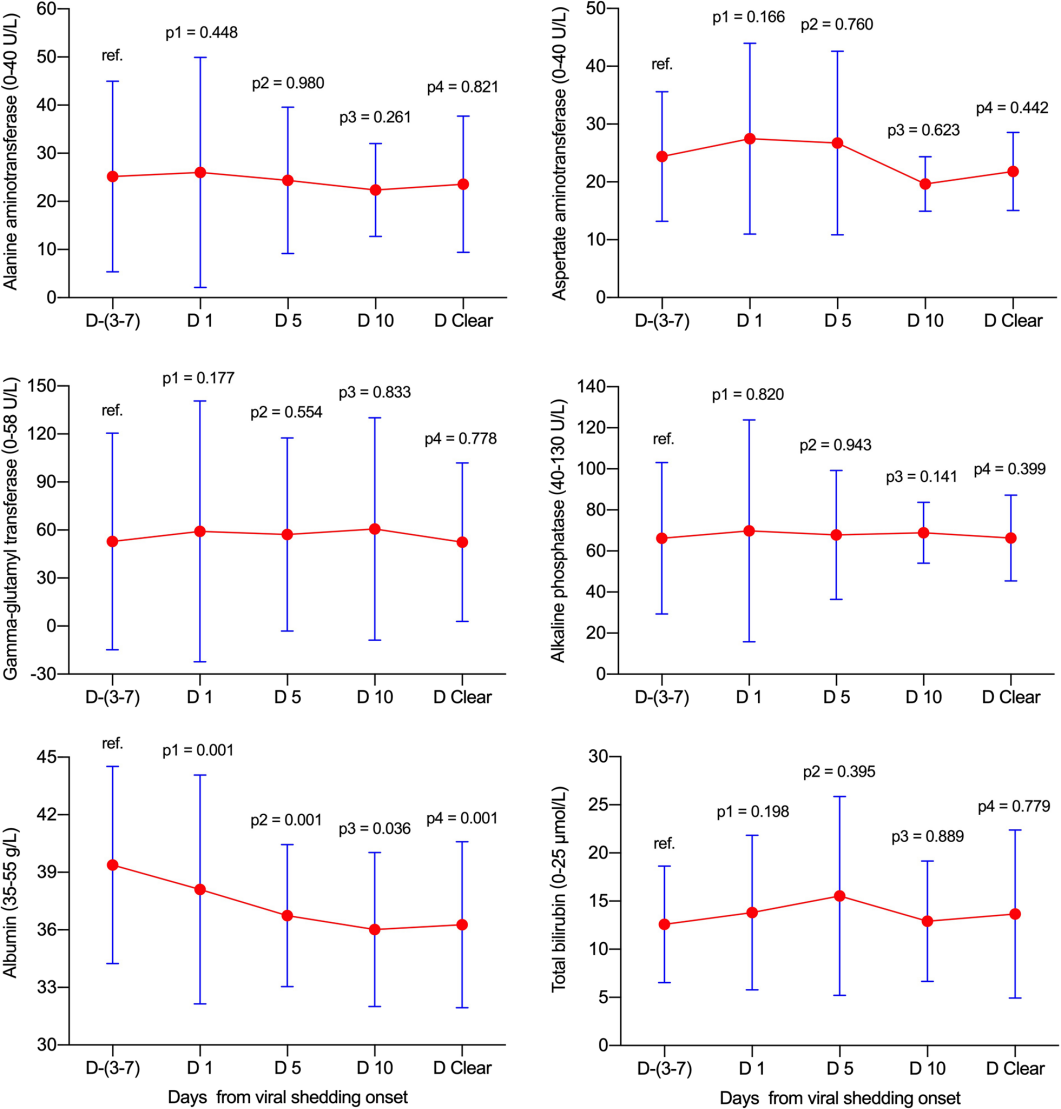
Dynamic levels of liver function test parameters in the non-ICU group. Data were presented as mean ± standard deviation. D −(3–7): n = 30; D 1: n = 30; D 5: n = 26; D 10: n = 8; D Clear: n = 30. D, day; ICU, intensive care unit; ref., reference

**Fig. 4:**
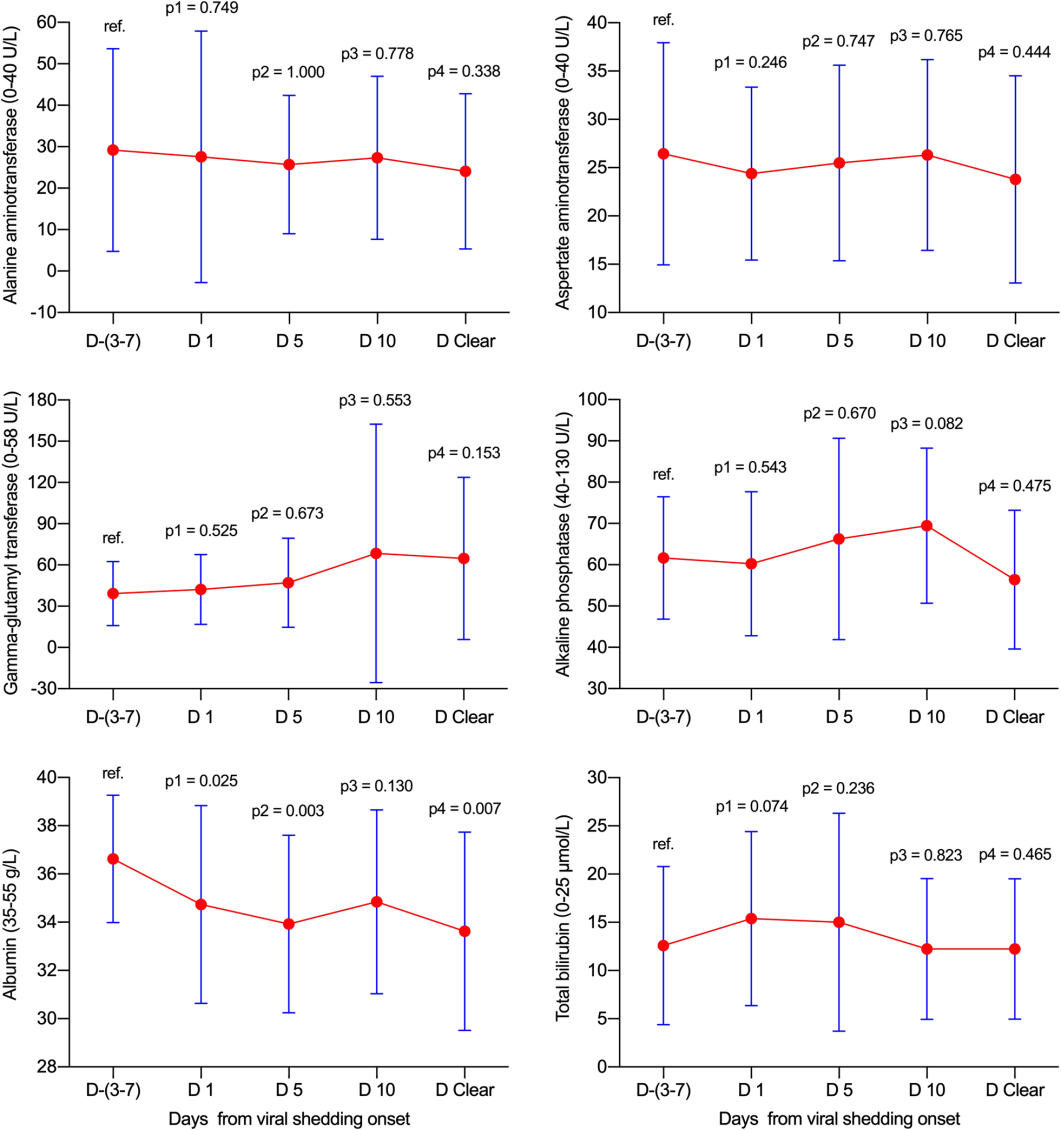
Dynamic levels of liver function test parameters in the ICU group. Data were presented as mean ± standard deviation. D −(3–7): n = 23; D 1: n = 23; D 5: n = 23; D 10: n = 20; D Clear: n = 23. D, day; ICU, intensive care unit; ref., reference

In the non-ICU and ICU groups, no significant elevations were observed for ALT, AST, GGT, ALP or TBIL at various time-points in reference to viral shedding (all *p* > 0.05, Fig. 3 and Fig. 4). Notably, the ALB levels decreased gradually from day −(3–7) to day clearance (all *p* < 0.05 with the exception of day 10 in the ICU group).

### Dynamic levels of liver function test parameters in the fatality group

**Fig. 5:**
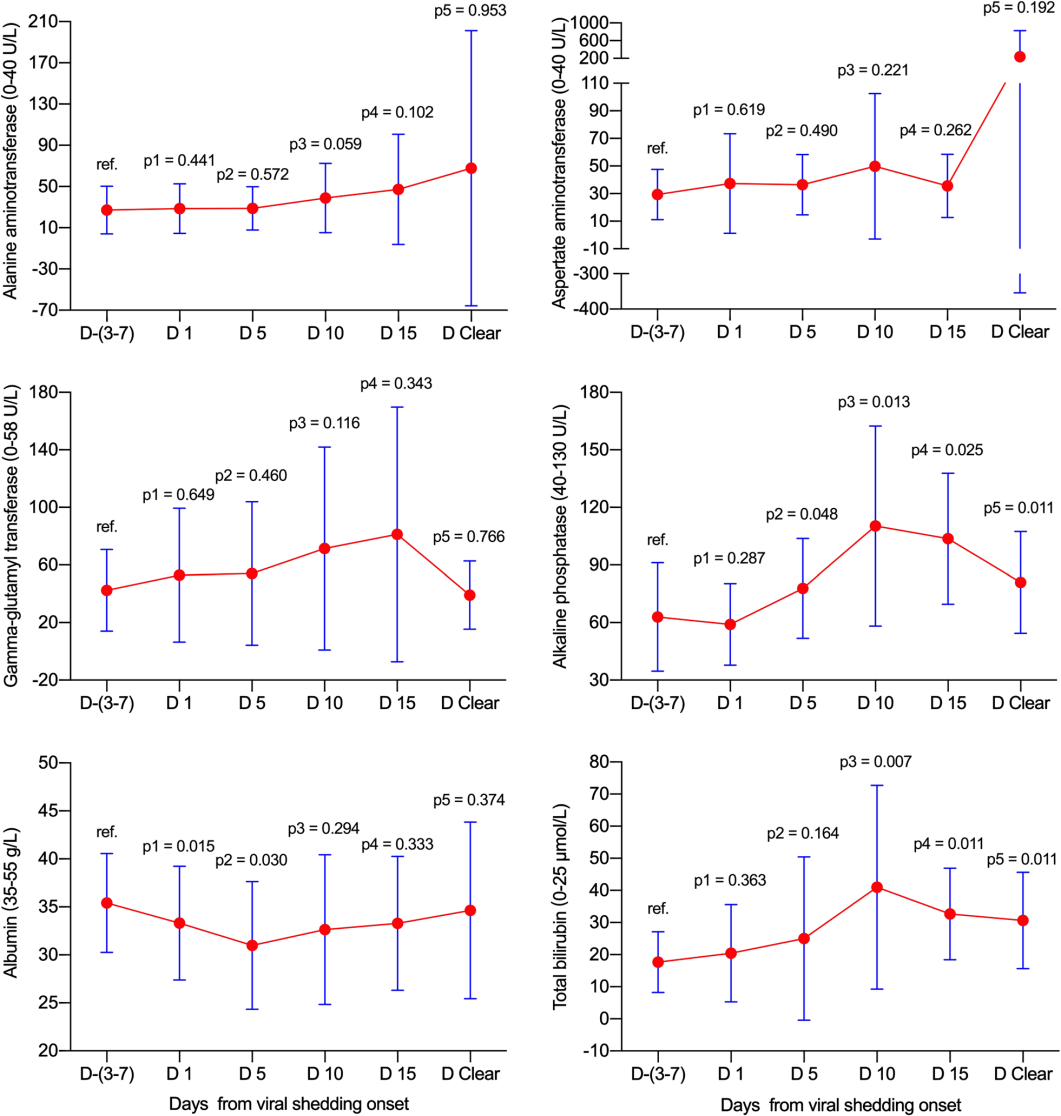
Dynamic levels of liver function test parameters in the fatality group. Data were presented as mean ± standard deviation. D −(3–7): n = 17; D 1: n = 17; D 5: n = 16; D 10: n = 13; D 15: n = 10; D Clear: n = 9. D, day; ref., reference

In the fatality group, no significant increases were found for ALT, AST or GGT at various time-points (all *p* > 0.05, Fig. 5). Meanwhile, ALP increased from day 5 (*p* = 0.048) to day clearance (*p* = 0.011), and TBIL increased from day 10 (*p* = 0.007) to day clearance (*p* = 0.011). Additionally, albumin decreased on days 1 (*p* = 0.015) and 5 (*p* = 0.030) during viral shedding.

### Dynamic changes in liver function test parameters in patients with chronic liver disease

**Fig. 6:**
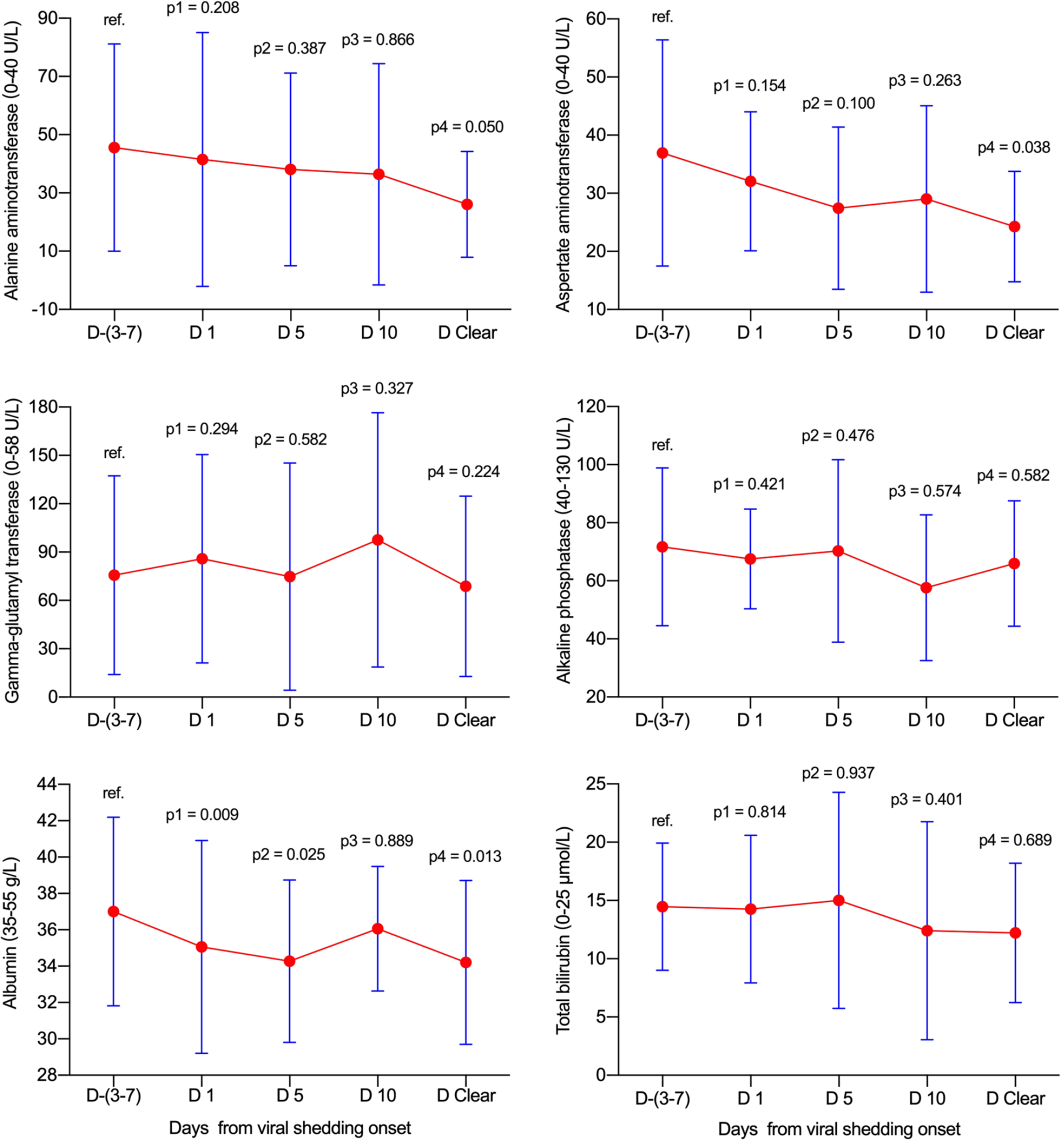
Dynamic levels of liver function test parameters in patients with chronic liver disease. Data were presented as mean ± standard deviation. D −(3–7): n = 13; D 1: n = 13; D 5: n = 12; D 10: n = 8; D Clear: n = 12. D, day; ref., reference

Two patients with chronic liver disease died due to non-liver-related reasons. The dynamic changes in liver function test parameters in 13 COVID-19 patients with previously diagnosed chronic liver disease are presented in Table 4 and Fig. 6. The ALT abnormal rates decreased unexpectedly and gradually from 38.5% (5/13) on day −(3–7) to 8.3% (1/12) on day clearance, and the ALT levels decreased simultaneously, although significant differences were not found (Fig. 6). Meanwhile, the AST abnormal rates and detailed levels had fluctuations similar to those in ALT, with the exception that a significant difference was observed for the AST level on day clearance (*p* = 0.038). Additionally, 30.8% (4/13) to 62.5% (5/8) of patients had GGT abnormal rates at various time-points, although no significant differences were observed. Furthermore, the abnormal rates of low albumin increased from 38.5% (5/13) on day −(3–7) to 58.3% (7/12) on day clearance, and the detailed levels also showed a decreasing tendency, although the difference was not significant on day 10 (*p* = 0.889). Notably, abnormal rates and levels for ALP and TBIL were steady during the full clinical course. For the 5 patients with chronic hepatitis B and 1 patient with chronic hepatitis C, no viral reactivations or breakthroughs were observed during hospitalization, and the liver function test parameters were also steady during the full clinical course of COVID-19.

**Table 4:** Dynamic changes in liver function test parameters in 13 COVID-19 patients with chronic liver disease

	Day − 3 to − 7	Day 1	Day 5^a^	Day 10^b^	Viral clearance^c^
ALT (0–40 U/L)	22 (19–76)	21 (15.5–67.5)	23.5 (14.8–73.8)	24 (11.5–54.5)	21.5 (17–28.3)
> 40 U/L	5 (38.5)	4 (30.8)	3/12 (25)	2/8 (25)	1/12 (8.3)
AST (0–40 U/L)	32 (20.5–45)	32 (23–39.5)	23 (17–34.8)	27 (15–41.3)	25 (14.8–30.8)
> 40 U/L	4 (30.8)	3 (23.1)	2/12 (16.7)	2/8 (25)	1/12 (8.3)
GGT (0–58 U/L)	50 (29–121)	61 (37–132)	42.5 (28.3–103)	78 (33–182.8)	50 (30–93.5)
> 58 U/L	4 (30.8)	7 (53.8)	4/12 (33.3)	5/8 (62.5)	4/12 (33.3)
ALP (40–130 U/L)	66 (57.5–69.5)	63 (54.5–81)	63 (46.3–85.5)	65.5 (37.5–75.8)	63 (52–70.5)
> 130 U/L	1 (7.7)	0 (0)	1/12 (8.3)	0/8 (0)	0/12 (0)
ALB (35–55 g/L)	35.1 (33.5–41.5)	34 (30–40.5)	32.8 (31.2–37.6)	36.7 (32.4–37.9)	33.2 (31.5–36.3)
< 35 g/L	5 (38.5)	8 (61.5)	6/12 (50)	3/8 (37.5)	7/12 (58.3)
TBIL (0–25 μmol/L)	15 (10.2–19.4)	10.4 (9.6–20.3)	12.5 (6.8–24.4)	8.5 (5.3–20.5)	10.7 (8.1–16.9)
> 25 μmol/L	0 (0)	0 (0)	3/12 (25)	1/8 (12.5)	0/12 (0)

Data are presented as median (interquartile range), n (%), or n/N (%), where N is the total number of cases with available data

^a^Data were unavailable in 1 patient because viral shedding discontinuation occurred on day 4

^b^Data were unavailable in 5 patients because viral shedding discontinuation occurred before day 10

^c^Data were unavailable in 1 patient because he did not clear the virus until death. The corresponding normal ranges and units of the liver function test parameters are presented in parentheses

## Discussion

SARS-CoV and the current SARS-CoV-2 have been demonstrated to be highly pathogenic human coronaviruses that can lead to respiratory, intestinal, hepatic, and neuronal diseases [15]. The current SARS-CoV-2 shares 79.6%-82% genome sequence similarity to SARS-CoV. It is known that SARS-CoV and SARS-CoV-2 employ ACE2 as the receptor for cell entry [16, 17]. ACE2 was reported to be abundantly expressed on endothelial cells of the liver, which makes the liver a potential target for SARS-CoV and SARS-CoV-2, although ACE2 expression is much higher on bile duct cells than on liver cells [9, 15]. Indeed, many studies have indicated that patients infected with human coronaviruses, not only the previous SARS-CoV [18, 19] but also the current SARS-CoV-2 [4, 5, 20–22], may have different degrees of hepatic impairment.

However, the relationship between SARS-CoV-2 and liver injury is still confusing because full liver function test parameters, dynamic changes in these parameters, and comparisons of these parameters referring to various timepoints of SARS-CoV-2 shedding were not included in previous studies [7, 26]. Commonly, viremia peaks in the first week after infection in most acute viral diseases, including SARS, and patients usually develop a primary immune response by days 10 to 14, which is followed by virus clearance [23]. In the third week, clinical deterioration occurs as the result of inflammatory or hyperimmune attacks rather than direct virus-induced tissue damage [23, 24]. Additionally, due to many unknowns during the early period of the COVID-19 pandemic in China and because the Chinese Government provided free full-course management of COVID-19 (from the first hospital visit to recovery), all suspected COVID-19 patients received biochemical tests (including liver tests) after their first visit to designated hospitals and were monitored during the process of COVID-19 until recovery or death in Henan Province, China, which provided important continuous data.

In the current study, 6 liver function test parameters were included. Notably, a total of 14% and 13% of patients had ALT and AST elevations on day −(3–7). Importantly, these abnormal rates did not significantly increase on day 1. More importantly, the detailed ALT and AST levels did not significantly fluctuate from day −(3–7) to day clearance in all groups, with the exception of AST abnormal rates that increased on day 5 and day clearance (median of 19 days) in the fatality group. These data indicate that ALT and AST elevations were not directly caused by SARS-CoV-2. The ALT and AST elevations before viral shedding may be associated with other factors. It was found that 13% and 31.4% of all the patients had previously been diagnosed with chronic liver disease and had a self-medication history after illness, respectively. The abnormal AST increase rates on day 5 and even on day clearance of viral shedding in the fatality group may be attributed to inflammation, hyperimmune attacks, or drug usage rather than directly to SARS-CoV-2-induced tissue damage because viral clearance may lead to discontinuation of injury, and the virus-related hyperimmune response and DILI may last longer, even after viral clearance.

As the diagnostic biomarker for cholangiocyte injury, the abnormal GGT rate significantly increased on day 10 (Table 2). Simultaneously, the GGT level significantly increased on day 10 (*p* = 0.033, Fig. 2). Meanwhile, the ALP abnormal rates did not show significant changes from day −(3–7) to the day of clearance of viral shedding (all *p* > 0.05), although the ALP levels were significantly elevated on day 10 and day clearance. Further analysis found that the fatality group contributed to the significant elevations in the ALP level (Fig. 5). These data indicate that SARS-CoV-2 may contribute to cholangiocyte injury. These results support the idea that SARS-CoV-2 employs ACE2 as the receptor for cell entry and leads to injury to the bile duct epithelium. It is worthy to note that few data are available to investigate the abdominal imaging of liver as well the bile ducts changes during the COVID-19 process. One preliminary report indicated that right upper quadrant CT or MRI examinations were less performed in COVID-19 patients, meanwhile, right upper quadrant ultrasound examinations were more performed because of liver laboratory findings (87%, 32 of 37), and 54% (20 of 37) revealed a dilated sludge-filled gallbladder, suggestive of bile stasis [25].

For TBIL (Table 2 and Fig. 2), the abnormal rates significantly increased on days 1, 5, and 10, and no significant difference was found on day clearance. Simultaneously, the TBIL level significantly increased on days 1 and 5, and no significant differences were found on day 10 and day clearance. Further analysis found that the fatality group contributed to the significant elevations in TBIL levels (Fig. 5). Notably, the majority of TBIL increases were attributed to direct bilirubin elevation, which indicates that the impairment of bile duct excretion and SARS-CoV-2 did not directly lead to impairment of hepatocytes.

Notably, the ALB abnormal decrease rates increased, and levels decreased significantly and simultaneously increased on days 1, 5, 10, and day clearance. These data strongly demonstrate that the synthetic function of the liver was severely affected. It is important to note that the severity degree of the ALB abnormal decrease rates and levels did not match the corresponding ALT and AST, which indicates that some other occult factors may be involved in decreasing ALB levels. It may be related to long-term consumption by disease and insufficient intake of nutrition and calories, or kidney impairment may be involved; future studies are needed.

Unexpectedly, the ALT and AST abnormal rates and levels were all decreased from day −(3–7) to day clearance in 13 patients with chronic liver disease (Table 4 and Fig. 6). This difficult-to-explain phenomenon may be caused by discontinuation of alcohol drinking after onset of COVID-19 illness because 5 of the 13 patients had previously been diagnosed with alcoholic liver disease and alcohol abuse. However, this phenomenon added clinical evidence to indicate that SARS-CoV-2 did not directly destroy hepatocytes. Additionally, no significant dynamic changes were found for GGT, ALP, and TBIL, with the exception of ALB decreasing on days 1 and 5 and day clearance. Interestingly, it is also hard to explain why the rate of the HBsAb/HBeAb/HBcAb-positive combination was significantly higher from the non-ICU to the ICU and fatality groups (Table 1). This may accompany the increase in age in the three groups because the HBV vaccine was unavailable and the HBV infection risk is higher in older age patients.

There are several limitations in the current study. First, we were unable to analyse the role of drug use (treatment) in the occurrence or deterioration of liver injury before and during hospitalization, although DILI during treatment of coronavirus infection may exist [6, 26]. Among symptomatic (fever, cough, et al.) patients with a self-medication history before admission, the majority (14/22) of them took traditional Chinese (herbal or patent) medicine; however, every one of these regimens included several (or even 10–15) herbs for only one dose; therefore, it is difficult to specify the medication component when the medical history is taken. Acetaminophen and levofloxacin/moxifloxacin were taken as instructed in a minority of symptomatic patients (8/22). Patients who took the medication at least once were counted as having met the criteria of “self-medication after illness” in this study; the longest treatment duration was one week. Additionally, due to the urgent situation during the beginning of the pandemic, the main focus was not alcohol consumption, and patients who had ever drunk alcohol were documented in the medical history, but we could not quantify the detailed intensity or duration from these available retrospective data. Notably, we indeed found more abnormal rates and levels of liver function test parameters in the fatality group than in the non-ICU and ICU groups, which may be partly caused by the higher number of drugs used in the fatality group. Second, serum cytokine levels could not be tested to analyse their potential role in liver injury because these data were unavailable for the majority of patients. The potential liver injury may also be caused by direct virus-induced cytopathic effects or immunopathology induced by overactive inflammatory responses, which may produce a large amount of cytokines and may be the cause of higher rates and levels of liver injury in the fatality group in the current study [15]. Third, the frequent serum albumin decrease in hospitalized COVID-19 patients should be interpreted cautiously because patients with other diseases requiring prolonged hospitalization may also show this result, and the absence of a control group may hinder a valid conclusion. Fourth, although all patients received liver function tests during hospitalization, we cannot completely exclude potential selection bias. In the current study, 243 patients were excluded (Fig. 1), 130 of whom were directly diagnosed on admission and therefore lacked the previral shedding liver function test data. Ninety of these patients were not tested for liver function parameters with a mean interval of 5 days, and we only retained patients with continuous testing from before viral shedding to recovery or fatality. Furthermore, all the fatal patients have ICU management and may overlap the survivals in ICU group, which could compromise the independency assumption for some multi-sample statistical tests. Finally, SARS-CoV-2 RNA quantitation was not available for patients during the beginning of the pandemic. Nevertheless, despite these limitations, our clinical judgement is that SARS-CoV-2 does not directly destroy hepatocytes because the ALT and AST abnormal rates and levels did not show any significant dynamic changes concurrent with SARS-CoV-2 shedding.

## Conclusions

To the best of our knowledge, this study is the first to utilize viral shedding as the reference to investigate the relationship between SARS-CoV-2 and liver injury and to present the dynamic changes in liver function test parameters before and during viral shedding and at the time of virus clearance in COVID-19 patients. In conclusion, we found that SARS-CoV-2 does not directly lead to elevations in ALT and AST but may cause elevations in GGT and TBIL (mainly direct bilirubin), which reflect impairment of the excretion function of the bile duct. Notably, the albumin levels were extraordinarily decreased, even when SARS-CoV-2 shedding was discontinued. Future large-scale, prospective validation studies for the relationship between SARS-CoV-2 infection and liver injury are needed.

## Data Availability

All data generated or analysed during this study are included in this published article.

## Abbreviations

ALB: Albumin
ALP: Alkaline phosphatase
ALT: Alanine aminotransferase
AST: Aspartate aminotransferase
COVID-19: Coronavirus diseases 2019
DILI: Drug-induced liver injury
CRRT: Continuous renal replacement therapy
GGT: Gamma-glutamyl transferase
HBcAb: Anti-hepatitis B core antibody
HBeAb: Anti-hepatitis B e antibody
HBeAg: Hepatitis B e antigen
HBsAb: Anti-hepatitis B surface antibody
HBsAg: Hepatitis B surface antigen
HBV: Hepatitis B virus
ICU: Intensive care unit
SARS-CoV-2: Severe acute respiratory syndrome coronavirus 2
TBIL: Total bilirubin
